# The HAPPY (Healthy and Active Parenting Programmme for early Years) feasibility randomised control trial: acceptability and feasibility of an intervention to reduce infant obesity

**DOI:** 10.1186/s12889-016-2861-z

**Published:** 2016-03-01

**Authors:** Rosemary R. C. McEachan, Gillian Santorelli, Maria Bryant, Pinki Sahota, Diane Farrar, Neil Small, Shaheen Akhtar, Judith Sargent, Sally E. Barber, Natalie Taylor, Gerry Richardson, Amanda J. Farrin, Raj S. Bhopal, Daniel D. Bingham, Sara M. Ahern, John Wright, on behalf of the BiB childhood obesity scientific group

**Affiliations:** Bradford Institute for Health Research, Bradford Teaching Hospital NHS Foundation Trust, Bradford Royal Infirmary, Duckworth Lane, Bradford, BD9 6RJ UK; Leeds Institute of Clinical Trials Research, University of Leeds, Leeds, LS2 9JT UK; Institute for Health and Wellbeing, Leeds Beckett University, Leeds, LS1 3HE UK; Department of Health Sciences, University of York, York, YO10 5DD UK; Faculty of Health Studies, University of Bradford, Bradford, BD7 1DP UK; Barnardo’s, Bradford, BD8 7BS UK; Australian Institute of Health Innovation, Macquarie University, Sydney, NSW 2109 Australia; Centre for Health Economics, University of York, York, YO10 5DD UK; Edinburgh Migration, Ethnicity and Health Research Group, Centre for Population Health Sciences, Institute of Population Health Sciences and Informatics, University of Edinburgh, Edinburgh, EH8 9AG UK; School of Sport, Health and Exercise Science, Loughborough University, Leicestershire, LE11 3TT UK

**Keywords:** Infant obesity, Behaviour change, Feasibility randomised controlled trial, Nutrition, Pregnancy, Ethnicity

## Abstract

**Background:**

The prevalence of infant obesity is increasing, but there is a lack of evidence-based approaches to prevent obesity at this age. This study tested the acceptability and feasibility of evaluating a theory-based intervention aimed at reducing risk of obesity in infants of overweight/obese women during and after pregnancy: the Healthy and Active Parenting Programme for Early Years (HAPPY).

**Methods:**

A feasibility randomised controlled trial was conducted in Bradford, England. One hundred twenty overweight/obese pregnant women (Body Mass Index [BMI] ≥25 kg/m^2^) were recruited between 10–26 weeks gestation. Consenting women were randomly allocated to HAPPY (6 antenatal, 6 postnatal sessions: *N* = 59) or usual care (*N* = 61). Appropriate outcome measures for a full trial were explored, including: infant’s length and weight, woman’s BMI, physical activity and dietary intake of the women and infants. Health economic data were collected. Measurement occurred before randomisation and when the infant was aged 6 months and 12 months. Feasibility outcomes were: recruitment/attrition rates, and acceptability of: randomisation, measurement, and intervention. Intra-class correlations for infant weight were calculated. Fidelity was assessed through observations and facilitator feedback. Focus groups and semi-structured interviews explored acceptability of methods, implementation, and intervention content.

**Results:**

Recruitment targets were met (~20 women/month) with a recruitment rate of 30 % of eligible women (120/396). There was 30 % attrition at 12 months; 66 % of recruited women failed to attend intervention sessions, but those who attended the first session were likely to continue to attend (mean 9.4/12 sessions, range 1–12). Reaction to intervention content was positive, and fidelity was high. Group clustering was minimal; an adjusted effect size of −0.25 standard deviation scores for infant weight at 12 months (95 % CI: −0.16–0.65) favouring the intervention was observed using intention to treat analyses. No adverse events were reported.

**Conclusions:**

The HAPPY intervention appeared feasible and acceptable to participants who attended and those delivering it, however attendance was low; adaptations to increase initial attendance are recommended. Whilst the study was not powered to detect a definitive effect, our results suggest a potential to reduce risk of infant obesity. The evidence reported provides valuable lessons to inform progression to a definitive trial.

**Trial Registration:**

Current Controlled Trials ISRCTN56735429

**Electronic supplementary material:**

The online version of this article (doi:10.1186/s12889-016-2861-z) contains supplementary material, which is available to authorized users.

## Background

Childhood obesity is a major global public health threat, [1, 2] with impacts on health and well-being lasting into adult life.[3–6] There is increasing evidence that risk of later obesity is established in early infancy, [7, 8] and thus this time period represents a potential period for intervention. Key modifiable factors in pregnancy and early infancy that are associated with childhood obesity include maternal overweight/obesity, [9, 10] maternal smoking, [10, 11] maternal diabetes, [8, 12] infant feeding patterns , [13] sleep duration, [8] sedentary behaviour and low physical activity, [8, 9, 14] and parenting and feeding styles [10]. There is thus the potential that interventions which tackle not only lifestyle related risk factors for infant obesity, but that also equip parents with suitable parenting skills might have benefit in reducing obesity.

Culture and ethnicity have a key role to play in development of obesity amongst children. Our previous research has found that infants of Pakistani origin, although on average lighter at birth, have more rapid growth in early infancy, which can be a risk factor for poor health in later child and adulthood [15]. Culture and ethnicity can also influence prevalence of obesity related risk behaviours. For example, we have also shown that White British mothers were more likely to have smoked during pregnancy, be obese, breastfeed their infants for a shorter duration, and display more indulgent feeding patterns than Pakistani mothers [10]. On the other hand, Pakistani mothers were more likely to report lower parental warmth and greater infant sedentary time. Taylor et al. [16] reported that barriers faced by these different groups in regards to changing these risk behaviours also vary. Frameworks for guiding efforts to adapt interventions to meet needs of ethnic minority groups are available [17, 18] and consideration of these issues are vital to ensure messages are presented in appropriate and relevant ways to target populations.

There is a dearth of evidence regarding effective interventions to reduce obesity targeted at infants. Notable exceptions include the Healthy Beginnings Randomised Controlled Trial [19] and the NOURISH trial [20]. The Healthy Beginnings trial found that infants of mothers randomly allocated to receive 8 dietary and lifestyle advices sessions delivered by community nurses until infants were 2 years of age had significantly lower BMI scores at 2 years than infants in the control group. In one of the only trials explicitly addressing parenting feeding practices, the NOURISH study found no significant differences in prevalence of overweight / obese infants at 2 years for mothers’ randomly allocated to receive 12 group sessions aimed at promoting protective feeding practices, but did see some positive effects on self-reported protective feeding behaviours [20]. Neither of these trials explicitly addressed issues of cultural adaptation for different groups in their development.

A major challenge for researchers in this area centres around the difficulties of recruitment and retention to obesity related trials. Reported recruitment rates from five recent antenatal trials targeting pregnant overweight women ranged from 20–40 % (MAMAS; [21] LIP; [22] UPBEAT; [23] NELLI; [24, 25] LIMIT [26, 27]). Recruitment rates for the NOURISH and Healthy Beginnings trials discussed above were 16 % and 24 % respectively. Attrition rates for these trials also varied with the Healthy Beginnings Trial reporting 20 % attrition at 12 months and 25 % at 24 months; and the Nourish Trial reporting 14 % at ~14 months and 24.5 % at ~24 months). MRC guidance for development and evaluation of complex interventions recommends researchers carry out feasibility and piloting of novel interventions in advance of definitive trials to explore issues around recruitment and retention, acceptability and to inform sample size calculations [28].

As part of a large programme of work exploring prospective risk factors for infant obesity, [10, 29] we recently reported the development of the HAPPY (Healthy and Active Parenting Programme for early Years) intervention – an antenatal and postnatally delivered intervention aimed at reducing infant obesity addressing key modifiable risk factors and the role of parenting [16]. The intervention was developed in the City of Bradford, UK, the 6^th^ largest city in the UK with high levels of deprivation, and an ethnically diverse population. The intervention was developed to be culturally appropriate for key groups within the city (White British and South Asian Origin women), and to specifically target overweight or obese pregnant women [16].

This feasibility study aimed to explore the acceptability of the HAPPY intervention to parents and service providers, and the feasibility of a phase III trial evaluation of HAPPY. The specific objectives of the feasibility study were to:

- Establish the recruitment rate and attrition rates for control and intervention groups and acceptability of the outcome measurement schedules in both groups
- Establish the acceptability of the intervention to parents, facilitators and service delivery partners
- Evaluate the fidelity of programme implementation and delivery by facilitators
- Estimate effect size and intra-class correlation co-efficients (ICC) for the primary outcome (infant obesity) to enable an accurate sample size calculation for a full trial

## Methods

### Study design

An individually randomised controlled feasibility trial with blinded assessment was used comparing the HAPPY programme with usual care. A process evaluation included (1) semi-structured interviews and focus groups with a total of 14 parents (across intervention and control groups), and 9 facilitators; (2) seven telephone interviews with women who were randomised to the intervention group but who did not attend any sessions; and (3) ten antenatal and five postnatal observations of intervention session delivery. The trial protocol was approved by Bradford Research Ethics committee on 8th February 2012 (Ref: 11/YH/0458). An independent trial steering committee including the head of midwifery, a statistician and parenting co-ordinator met quarterly to oversee progress.

### Participants

Inclusion criteria were: Overweight and obese pregnant women (Body Mass Index: BMI of ≥25 kg/m^2^, at time of registering their pregnancy, typically between 8–12 weeks gestation); at least 18 years of age; able to attend intervention sessions (e.g. able to travel to venue), attending antenatal appointments at the Bradford Royal Infirmary Women’s and Newborn Unit (BRIWNU), Bradford, England. Exclusion criteria included pre-existing self-reported serious physical or mental health disorder, a known fetal abnormality or a multiple pregnancy. Due to resource constraints we were only able to include women who could understand intervention sessions delivered in English.

### Study settings and recruitment

Screening and recruitment took place at the BRIWNU by research midwives and research administrators. Around 49 % of women attending the unit are of South Asian origin [30]. Over half of the women attending this unit are classed as overweight or obese (BMI ≥25) at the time of registering their pregnancy [31]. Screening for eligibility was conducted at either the 10–12 week dating scan or the 20 week normality scan by research midwives or research administrators. Eligible women were subsequently contacted by a researcher to discuss participation.

For eligible consenting women, data were collected by researchers within family homes at baseline (prior to randomisation, ~22–26 weeks gestation) and when the infant was aged 12 months. An interim data collection assessment was collected via telephone when the infant was aged 6 months.

### Randomisation

Eligible participants were randomised to intervention or control on a 1:1 ratio by Leeds Clinical Trials Research Unit using a minimisation algorithm incorporating a random element, stratified by maternal body mass index (BMI: 25–29.9 kg/m^2^ / ≥30 kg/m^2^), ethnicity (South Asian/Other) and parity (first child/at least one other child). A secure centralised telephone based service was used to randomise participants, using a concealed computerised random allocation sequence generated by independent trial statisticians. Randomisation occurred immediately following the baseline assessment.

### The intervention

HAPPY was developed in partnership with the Family Links Nurturing Antenatal Programme (FLNP) (https://familylinks.org.uk/what-we-do). It consists of a range of verbal and written advice and activities delivered by parenting facilitators in a group setting to target specific behaviours in the mother that if adopted might prevent obesity in their child, and also to promote positive parenting skills in recognition of the relationship between aspects of parenting and obesity in their children (e.g. [32, 33]), this includes promotion of a healthy lifestyle (e.g. physical activity, healthy diet), for both mother and infant. A detailed manual is available for facilitators which sets out content, timetable and instructions for all components of the intervention [16].

HAPPY is delivered in 12 group sessions (6 antenatal, 6 postnatal), with overall aims to:

1. Encourage the mother to make healthy food choices antenatally and maintain a healthy diet postnatally
2. Encourage the mother to increase physical activity during pregnancy and meet the recommended guidelines (150 min moderate intensity physical activity per week, [34]) within 12 months of giving birth
3. Encourage breast (or bottle) feeding until at least 6 months of age
4. Encourage the infant to develop healthy food preferences and dietary intake
5. Facilitate infant physical activity and limit sedentary time

A full description of the intervention, including the rationale underlying each of the aims can be found in [16], and a summary of the key elements and logic model can be found in Additional file 1. The intervention is underpinned by behavioural theory and targets key determinants of health behaviours identified by Michie *et al.,* [35] (for example, knowledge, motivation, social norms, skills and competencies) and uses standardised behaviour change techniques (for example, setting ‘graded tasks’, modelling of appropriate behaviour) [36]. The parenting aspects of the intervention, developed by FLNP, aimed to increase self-awareness and self-esteem, manage expectations, and promote empathy and positive discipline [37–39]. The intervention was developed with reference to a cross-cultural typology of adaptation approaches to ensure it was sensitive to ethnic minority groups [17 18]. Cultural appropriateness for both White British and South Asian groups was ensured through 1) the use of community resources to develop and publicise the intervention, 2) identifying and addressing barriers to access and participation, 3) developing communication strategies which were sensitive to language and information requirements, 4) consideration of cultural/religious values that promote or hinder behavioural change, 5) recognising degrees of ethnic identification [40]. Additionally local practitioners (e.g., dieticians, infant feeding advisors, parenting practitioners, community health workers) with a wealth of experience in delivering community based interventions to a range of ethnic groups informed the intervention development. The intervention was then mapped across a theoretically underpinned typology for cultural adaptation of interventions designed to maximise the cross-cultural appropriateness and effectiveness of health promotion interventions within South Asian-origin populations [17].

Antenatal sessions were planned weekly starting when the woman was around 26–28 weeks pregnant, and contained women with similar estimated dates of delivery (+/− 4 weeks of one another). Each session lasted approximately two and a half hours. Postnatal sessions (which both mother and infant attended) started when the infant was aged four to 6 weeks and continued at key developmental milestones up to 9 months. Sessions were delivered in community locations (e.g. Children’s Centres) by existing FLNP facilitators, for whom additional training was provided. For the purposes of the research, participants were reimbursed expenses associated with attending the intervention sessions (£15 per session).

### Comparator

Participants in the Control group received usual care. In Bradford this included access to and support from health professionals and support agencies including midwives, obstetricians health visitors, general practitioners and self-accessed services delivered in a range of locations (e.g. children’s centres, health clinics, voluntary sector provision). Women in the active intervention arm had access to these services in addition to the HAPPY intervention.

### Measures

At baseline, data were collected on socio-demographic variables: ethnicity, marital status, cohabitation details, country of birth, age moved to UK (if not born in UK), education level and smoking status. Women’s height and weight at the time of ‘booking’ (the first contact with midwifery services, around 8–12 weeks gestation) were collected from maternity notes in order to calculate BMI at baseline. Infants’ birth weights were obtained from birth records. Infant weight and length was measured during a home visit at 12 months follow-up by trained research administrators. Additional measures included maternal diet [41] and BMI, home food environment, [42] physical activity, [43] parenting practices, [44] and infant diet [45], physical activity and development [46]. Validated or objective measures were used where they were appropriate and available. Where no validated tools were available, study specific measures were used. A summary of all measures is presented in Table 1, with further information in Additional file 2.

**Table 1:** Details of outcome measures used within feasibility study

Outcome measure	Detail of measure	Baseline (home visit)	6 months (telephone interview)	12 months (home visit)
Objective measures				
Mother’s BMI	Measure of height (baseline only, collected from maternity notes) and weight (using calibrated SECA [model 877] scales )	✓		✓
Child’s weight and length	Weight (measured using calibrated SECA [model 877] scales at 12 months)	✓	✓	✓
	Length measured using rollameter	Birth weight from hospital records	Self-reported from health visitor assessment	Assessed by interviewer
Maternal diet				
Maternal diet	Fruit and vegetable consumption: FACET [41]	✓		✓
Environment	Home food availability inventory [42]	✓		✓
Infant diet				
Breastfeeding/weaning	Duration of breastfeeding and age at weaning^a^		✓	✓
Infant diet	Food Frequency Questionnaire – Infant [45]			✓
Maternal physical activity				
Maternal physical activity	7 day physical activity recall (self-report) [43]	✓		
	Accelerometer worn on waist (anterior to the iliac crest) for 7 days			✓
	(GT3X Actigraph accelerometer)			
Maternal sedentary behaviours	Sitting Time Questionnaire [70]	✓	✓	✓
Infant physical activity				
Infant physical activity	Tummy time questions [71]		✓	
	Sitting questions^a^			✓
	Accelerometer worn on waist (anterior to the iliac crest) for 7 days			✓
	(GT3X Actigraph accelerometer)			
Maternal parenting practices/feeding styles				
Parenting practices	Parenting practice questionnaire [44]			✓
Infant feeding styles	Infant feeding questionnaire [72]			✓
Infant development				
Infant development	Ages and Stages (gross motor skills) [46]		✓	✓
			(by post)	

^a^Denotes items developed specifically for current study (see Additional file 2 for details)

At baseline, a commonly used seven day ‘physical activity recall’ measure was used to assess women’s physical activity; [43] however, many participants and assessors expressed that it was burdensome to complete. The questionnaire was therefore replaced with an objective measure of physical activity using activity monitors during the 12 month follow-up assessment for both women and infants. This is consistent with recent guidance on use of objective measures of physical activity as outcomes for children’s obesity intervention [47, 48].

At 12 months, mother and infant pairs were asked to wear a tri-axial accelerometer (Actigraph GT3X+; Actigraph Pensacola) around the waist (anterior to the iliac crest) during waking hours for 7 days. Data were downloaded and reduced using Actilife software version 6. Mothers’ raw Actigraph GT3X+ files were converted into 60-s epochs. Time spent sedentary (0–99 counts), in light physical activity (LPA) (100–759 counts) and in moderate-to-vigorous physical activity (≥1952) were calculated using the standard cut points [49]. Non-wear time was defined as ≥60 min of consecutive zeros. Minimum wear time was calculated using the Spearman-Brown prophecy formula [50], which resulted in a value of seven hours on any three days (please see Additional file 3 for further details of this analysis). Infants’ raw Actigraph GT3X+ files were converted into of 5-s epochs and non-wear time was defined as ≥10 min of consecutive zeros. Accelerometers have not previously been calibrated or validated in children under the age of 1.5 years; therefore there are no calibrated cut-points to determine counts required to reach different physical activity intensities. Because of this, the vector magnitude counts per minute (CPM) was used as a measure of total physical activity for infants. The vector magnitude takes into account all three axis of the accelerometer and increases the likelihood of capturing infant movements such as crawling. Minimum wear time for infants, calculated using the Spearman Brown prophecy formula [50] was five hours on any two days (please see Additional file 3 for full details of this analysis).

### Fidelity measures and acceptability measures

Facilitators completed a brief structured feedback questionnaire after each session asking a) ‘how did you find the delivery of the content of this session’ (rated on a 5 point Likert-type scale where possible answers were 1: complicated/challenging to 5: straightforward/easy, and b) ‘how well did the group engage with the materials/concepts’ (1: not at all to 5: very receptive). Facilitators also noted any changes to content delivery, comments on timings, materials (for example content and hand-outs), and general thoughts or feelings about the session.

In addition, 15 direct observations were conducted by the parenting programme coordinator. Facilitators were rated on whether they delivered components of the sessions according to the manual as follows: 1: no elements of the programme delivered; 2: some elements of the programme, others missed or inserted; 3: majority elements of the programme delivered; 4: all elements of the programme delivered as per handbook.

After each session, parents completed a brief questionnaire. They were asked to rate ‘how was today’s session for you’ on a 5 point scale (1: awful to 5: great). Open ended questions asked them to indicate what was a) most useful; b) their favourite thing; c) least useful; d) their least favourite thing about the session. Space was provided for other comments or suggestions. Acceptability was further explored within semi-structured interviews and focus groups.

### Sample size

As this was a feasibility study, a formal sample size calculation to evaluate effectiveness was not appropriate. The aim was to recruit 120 participants and randomise 60 to the intervention, and 60 to the usual care. This would provide the intervention arm with six groups of 10 women, with similar estimated delivery dates, geographically spread to the north and the south of the city.

### Blinding

We attempted to conduct outcome assessments using researchers who were blind to the group participants had been randomised to. However, blinding of those collecting data was only partially successful as participants often spontaneously referred to their experience of attending intervention sessions during follow-up assessments. We therefore refer to this as partial blinding of outcome assessment. All analyses were conducted by a statistician blinded to group allocation. It was not possible to blind participants, or those delivering the intervention.

### Analytical methods

Descriptive statistics were used to explore recruitment, retention and attrition rates for the feasibility trial. Multivariable logistic regression was used to explore factors related to consenting to participate in the study, and multivariable linear regression examined factors associated with attendance at intervention sessions. Explanatory variables for both regressions included ethnicity (Pakistani, White British, Other); parity (continuous variable, range 0–10); booking BMI; gestational age at screening (calculated as 40 weeks – (estimated delivery date – screening date)), and maternal age at screening. Key baseline and follow-up data were summarised using mean (standard deviation (SD), or 95 % confidence intervention (95 % CI)) or median (inter-quartile range (IQR)) for continuous variables and frequencies and proportions for categorical variables. Field notes collected during interviews and focus groups, and free text responses from facilitator and participant feedback forms were analysed using a thematic analysis approach (as outlined in [51]) by RM, SA and NS.

### Intended definitive trial outcomes

In order to determine an appropriate primary outcome for the definitive trial (for example, weight, rapid growth, or prevalence of overweight) weight data were converted to age and sex-adjusted standard deviation scores (SDS) using the World Health Organization (WHO) 2006 growth standard [52]. Infant weight was examined at age 12 months as a continuous variable. We also created two ‘conditional’ weight gain variables using the SDS: (1) the proportion of infants who crossed one centile band for weight between birth and 12 months (weight change SDS >0.67); and (2) the proportion of infants who crossed two centile bands for weight between birth and 12 months (weight change SDS >1.33). Finally, the proportion of overweight infants with weight > 85^th^ centile at 12 months was calculated.

To estimate potential effect size, a number of linear and logistic regression models were conducted for each outcome using intention to treat analyses. Model (A) was an unadjusted regression model to examine the relationship between randomised group and weight. Model (B) adjusted for the stratification factors (maternal BMI, parity and ethnicity). As it was not planned to stratify by ethnicity in the definitive trial, model (C) adjusted only for maternal BMI and parity. Multilevel modelling (model D) was used to calculate the intra-cluster correlation coefficient (ICC) to account for clustering in the intervention and control groups. As this was a feasibility study and therefore not designed to measure effectiveness it was inappropriate to impute missing outcome data, thus these analyses excluded participants with missing child weight at 12 months.

## Results

### Participant flow and recruitment

**Fig. 1:**
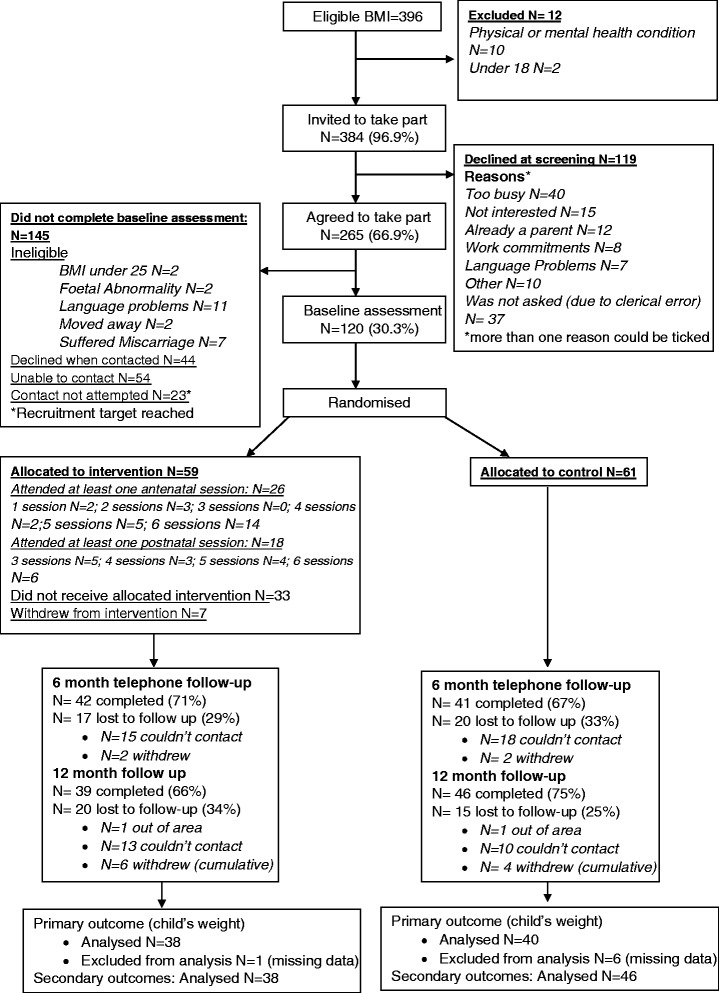
Participant flow through the study

Figure 1 shows the flow of participants through the trial. One hundred and twenty women were recruited to the study between 5^th^ March 2012 and 8^th^ November 2012. After baseline assessment, 59 participants were randomly allocated to the intervention group and 61 were allocated to the control group. Follow up occurred when infants were aged 6 months (median 6.14, IQR: 5.84–8.11) between March 2013 and September 2013, and 12 months (median 12.22, QR: 11.99–12.64) between July 2013 and March 2014.

Based on their medical records, 396 women were identified as having an eligible BMI (≥25) at the time of booking; of these, the overall recruitment rate (i.e. consenting to participate) was 30 %. At screening 384 met the inclusion criteria and 69 % agreed to be contacted to ascertain if they wished to take part (*N* = 265). Reasons for refusal are detailed in Fig. 1. Main reasons included being too busy or not being interested.

Table 2 shows baseline characteristics of the 120 participants, overall and by randomised group. Women’s mean BMI at booking was 29.7 (SD 4.8, range 25.0 to 47.5). Thirty per cent (*N* = 40) of women agreeing to take part were primiparous and 82 % (*N* = 99) were married or cohabiting with a partner. Forty-six per cent (*N* = 55) of women were of Pakistani origin with 29 % (*N* = 35) of White British origin. Sixty five per cent (*N* = 78) of the sample were born in the UK.

**Table 2:** Baseline characteristics of sample. Values are frequency (%) unless otherwise stated

Variable	All	Intervention	Control
	*N* = 120	*N* = 59	*N* = 61
Socio-demographic			
Woman’s BMI: Mean (SD)	29.7 (4.8)	29.5 (4.7)	30.0 (4.9)
Missing	3 (2.5)	1 (1.6)	2 (3.3)
First child	36 (30.0)	20 (33.9)	16 (26.2)
Marital status			
Married/cohabiting	99 (82.5)	46 (78.0)	53 (86.9)
Single/lives alone	21 (17.5)	13 (22.0)	8 (13.1)
Ethnicity			
Pakistani	55 (45.8)	29 (49.2)	26 (42.6)
White British	35 (29.2)	19 (32.2)	16 (26.2)
Other	28 (23.3)	11 (18.6)	17 (28.9)
Missing	2 (1.67)	0	2 (3.3)
Country Woman born in			
UK	78 (65.0)	41 (69.5)	37 (60.7)
Pakistan	21 (17.5)	10 (17.0)	11 (18.0)
Other South Asian country	8 (6.7)	4 (6.8)	4 (6.6)
Other country	13 (10.8)	4 (6.8)	9 (14.8)
Age moved to UK (if not born in UK)	18.1 (8.9)	14.3 (10.0)	21.0 (6.9)
Woman’s education			
< 5 GCSEs	18 (15.0)	7 (11.9)	11 (18.0)
5+ GCSEs	37 (30.8)	21 (35.6)	16 (26.2)
A level	21 (17.5)	12 (20.3)	9 (14.8)
Degree level	37 (30.8)	17 (28.8)	20 (32.8)
Other	7 (5.8)	2 (3.4)	5 (8.2)
Smoked during pregnancy	20 (16.7)	10 (17.0)	10 (16.4)
Maternal physical activity (sitting time)			
Hours spent sitting each week day: Median (IQR)	4.7 (3.0, 7.5)	4.0 (3.0, 7.3)	5.2 (2.9, 8.0)
Hours spent sitting each weekend day: Median (IQR)	5.0 (3.0, 7.0)	4.8 (2.7, 7.0)	5.5 (3.3, 7.5)
Maternal physical activity recall (PAR)			
Energy expenditure per day (calories): Mean (SD)	2623 (461)	2579 (463)	2672 (459)
Missing	20	6	14
Maternal diet			
Daily fruit and vegetable intake (portions): Median (IQR)	6.0 (4.0, 9.0)	6.0 (4.0, 9.0)	6.0 (4.0, 8.0)
Missing	0	1 (1.6)	1 (0.8)
Foods present in the home (% present)			
Fruit	118 (98.3)	58 (98.3)	60 (98.4)
Vegetables	119 (99.2)	59 (100.0)	60 (98.4)
Snacks	115 (95.8)	54 (91.5)	61 (100.0)
Fizzy drinks (exc. diet drinks)	101 (84.2)	48 (81.4)	53 (86.9)
Quantity of foods present in the home (portions present)			
Fruit: Mean (SD)	6.8 (3.1)	6.4 (2.6)	7.2 (3.5)
Vegetables: Mean (SD)	6.4 (2.8)	6.3 (2.9)	6.6 (2.7)
Snacks: Mean (SD)	3.9 (1.8)	3.8 (1.9)	4.0 (1.9)
Fizzy drinks (exc. diet drinks): Mean (SD)	1.5 (0.2)	1.4 (1.0)	1.4 (1.0)

Those who initially expressed an interest in taking part, but did not agree to participate or complete a questionnaire, had a similar BMI to those recruited (mean 30.0, SD 4.8). Logistic regression analysis of those who agreed to be contacted regarding participation (excluding those deemed ineligible *after* initial screening, and those who were not contacted as the recruitment period ended, see Fig. 1, *N* = 189 with complete data) confirmed that White British women were less likely to enrol in the study compared to Pakistani women (Odds Ratio 0.43, 95 % CI: 0.22, 0.85, *p* = 0.015, full details in Additional file 4). Enrolment status was not associated with maternal age, gestational age at screening, parity or BMI at booking.

Six women within the intervention group and four women within the control group withdrew from the trial. Reasons included being too busy, no longer interested, competing family commitments, or ill-health. Attrition rate at 6 months was 30.8 % (83 women completing the assessment), and was similar between treatment arms. At 12 months, 85 women completed the assessment (total attrition 30.2 %); with 34 % loss to follow-up in the intervention arm (*N* = 20) and 25 % loss in the control arm (*N* = 15).

### Acceptability of randomisation strategy

There were mixed reactions to the randomisation strategy. Many women said they were unsure about why they had been approached to take part in the study and some said they did not realise the intervention was aimed at overweight/obese women. Few of the women interviewed understood the randomisation process. Intervention women were happy to have been selected and discussed ‘feeling privileged’ and talking about their excitement about being able to join the ‘club’ (in reference to HAPPY antenatal sessions). Some control group women interviewed expressed disappointment at being allocated to the control group.

### Acceptability of measurement tools

Data from measurement tools at baseline can be found in Table 2. Data from the 6 and 12 month follow-up assessments are available in Additional file 4. The baseline assessment took around 75 min and participants reported that the food and physical activity sections were particularly burdensome (16 % of participants did not complete the physical activity survey at baseline). Telephone interviews at 6 months took 10–15 min to complete. Women were asked to report the last weight/height recorded by a health professional in their infant’s health record: 18.1 % (*N* = 15) reported length being available (median month of measurement 2.2, IQR: 0.9–3.9), and 68.7 % reported weight as being available (median month at measurement 2.0 (IQR: 1.0–2.9). Response rates for return of the ages and stages questionnaire which was sent via post was 38.5 %.

At 12 months, research administrators failed to collect weight for three women, and weight and length for seven infants. Reasons included infant or woman being ill or infant being asleep at time of visit. The follow-up assessment took approximately 75 min. Accelerometer belts were given to 78 women and infant pairs (92.2 % of those attending 12 month follow-up) who were instructed to wear the accelerometers daily over the coming week. All accelerometers were returned after 1 week. Of these, 27 women (35.5 %) wore the accelerometer for an acceptable wear time of 7 h per day for 3 days (see Additional file 3). Seventy-three women (85.8 % of those attending 12 month follow up) accepted an accelerometer for their infant. Of these, 33 infants (45.0 %) wore the belt for an acceptable wear time (5 h on 2 days, see Additional file 3).

All participants except one (at the 6 month follow-up) completed the modified cost questionnaire assessing primary and secondary care use at both 6 and 12 months, and the EQ-5D measure of quality of life at 12 months.

### Effect sizes and determination of primary outcome measures

At 12 months, infants in the intervention arm were 0.33 SDS above the average weight according to WHO guidelines (95 % CI: 0.02, 0.64), whilst infants in the control arm were heavier at 0.53 SDS above the average (95 % CI: 0.24, 0.81). Twenty three per cent of infants within the intervention group were classed as overweight and crossing one centile band compared with 45 % within the control. Eleven per cent of infants in the intervention group and 20 % of infants in the control group crossed two centile bands between birth and 12 months. Eighteen per cent of the intervention weighed greater than the 85^th^ centile compared with 25 % of the control group.

Table 3 presents the coefficients or odds ratios (OR) for the difference between the intervention and control arms. The unadjusted effect size (model A) for weight SDS between the two groups was −0.20 (95 % CI −0.62, 0.21), favouring the intervention. Adjustment for stratification factors (models b and c) increased the effect size to −0.25 (95 % CI: −0.65, 0.16 for model b, and −0.66, 0.16 for model c). The ICC was exceptionally small (1.96^−24^).

**Table 3:** Effect sizes for infant weight at 12 months

Outcome	Model a	Model b	Model c
Age and sex-adjusted weight SDS at 12 months^a^			
Control arm	0.00	0.00	0.00
Intervention arm	−0.20 (−0.62, 0.21)	−0.25 (−0.66, 0.16)	−0.25 (−0.65, 0.16)
Conditional weight gain > 1 centile band^b^			
Control arm	1.00	1.00	1.00
Intervention arm	0.38 (0.14, 1.00)	0.29 (0.10, 0.84)	0.29 (0.10, 0.85)
Conditional weight gain > 2 centile bands^b^			
Control arm	1.00	1.00	1.00
Intervention arm	0.47 (0.13, 1.72)	0.39 (0.10, 1.52)	0.38 (0.10, 1.49)
Weight >85^th^ centile at 12 months^b^			
Control arm	1.00	1.00	1.00
Intervention arm	0.68 (0.23, 2.01)	0.48 (0.14, 1.62)	0.50 (0.15, 1.64)

Model a –unadjusted; Model b – adjusted for all stratification factors (maternal BMI, parity and ethnicity); Model c - adjusted for maternal BMI and parity; . ^a^Mean (95 % CI); ^b^OR (95 % CI)

### Determination of a sample size for the definitive trial

In order to determine the sample size for the definitive trial it was necessary to determine a) length of follow-up for measurement of the primary outcome, b) the ‘minimum clinically important difference (MCID)’ we would wish to see as a result of the intervention and c) an appropriate intra-class correlation co-efficient. In order to make these decisions we used evidence from our recent programme of research exploring the epidemiological predictors of obesity in early infancy (the BiB1000 study [29]) and also from a review of similar trials aimed at tackling childhood obesity in early years [53]. After reviewing this evidence we first decided that for the definitive trial, the primary outcome would be measured at 24 months to allow any natural fluctuations in weight loss and catch-up to be minimised, and to provide a greater opportunity to assess the impact of the intervention on obesity. Using this endpoint, we would be able to calculate BMI z-scores, which are not possible at 12 months due to a lack of reference data, and compare our findings to other similar trials [19, 20].

We found no clear guidance upon which to base our decisions regarding our MCID. For example, National Institute for Health and Care Excellence (NICE) guidelines [54] state that one should expect BMI z-score differences of 0.2 for lifestyle weight management *treatment* programmes for children, but there are no standards on what is considered clinically meaningful for obesity *prevention* in preschool children. We contacted NICE to discuss this and have been told that during the guidance development, the available evidence for this age group was weak, with outcomes focused “on psychological well-being rather than clinical indicators like blood pressure or lipids for example” (NICE, personal communication, 2015). Within the UK, we located a *preventive* trial targeted at older children aged 6–7 years (http://www.nets.nihr.ac.uk/projects/hta/068511) which had been powered to detect at MCID of 0.25 BMI z-scores. This was based on data from Ford et al. [55] in which a reduction of 0.25 BMI z-score led to improved insulin sensitivity, total cholesterol/high-density lipoprotein ratio and BP in obese adolescents; although, again, this related to obesity treatment and not prevention. We obtained guidance from a range of clinical experts who agreed that a reduction of 0.25 SDS (~400 g) would be the minimum acceptable difference for the HAPPY trial, when considering outcomes at 24 months.

Similarly, there is scant information from previous trials regarding an appropriate ICC. The ICCs from this feasibility trial were negligible, and of the few trials in this area, many fail to report ICCs. Campbell et al., [56] examined ICCs within 21 datasets of group interventions delivered in primary or secondary care in the UK. The median ICC within these datasets for outcomes and process measures was 0.048; however, this was highly skewed (16.8 % were censored at 0). ICCs within primary care (median 0.045) were lower than those in secondary care (0.061) and clusters within sub-units of GP practices (median ICC, 0.01) were lower than those within whole GPs (0.048) or hospitals (0.054). Data from this study was used to power a weight management trial in pregnancy (HELP, [57]) in which an ICC of 0.02 was included. The WAVES trial identified above also included an estimated an ICC of 0.02 [58]. Based on those studies, we all chose an ICC of 0.02 to inform the sample size calculation for a full trial, which we felt was conservative, given the negligible ICC apparent in the current feasibility trial.

Assuming equal treatment group allocation, ten participants per cluster, 90 % power and 5 % significance (two-tailed), a sample size of 1080 participants (540 per arm) would allow the detection of a minimum clinically important difference of 0.25 BMI (kg/m^2^)SDS at 24 months, incorporating a maximum loss of follow-up of 25 % and an ICC of 0.02.

### Acceptability of the HAPPY intervention

On average, women in the intervention group attended 2.19 antenatal sessions (SD 2.66), and 1.41 (SD 1.43) postnatal sessions. Twenty six women (44 %) received at least one antenatal intervention session, and 21 (36 %) received four or more antenatal sessions (mean attendance 4.8 sessions), with 14 (24 %) attending all six sessions. Of the 26 women who attended at least one session, the mean attendance rate was 4.8 sessions antenatally. Eighteen women attended at least three postnatal sessions (30.5 %), and 13 attended four or more (22.0 %), mean attendance 4.6 sessions). The mean number of attendees per group was four in the antenatal sessions and 3.5 in the postnatal sessions. A linear regression analysis (*N* = 53 with complete data) indicated there was no association between attendance at any antenatal intervention session and maternal age, gestational age, parity, booking BMI or ethnicity (see Additional file 4).

Parent feedback from those who attended the intervention, assessed at the end of each intervention session was positive (mean rating 4.70/5, standard deviation 0.47, from 151 completed parent feedback forms), with very few negative responses to the intervention elicited (centring around problems with the venue). Practical issues were important in facilitating attendance, and a future trial should aim to ensure access to facilities such as crèche and car-parking. One issue for both participants and facilitators was setting up the groups in a convenient location. Some participants reported making journeys that required considerable effort (such as changing buses, going to unfamiliar parts of the city), which was problematic. Feedback from those women allocated to the intervention, but who subsequently did not attend highlighted the importance of ensuring a realistic expectation of the commitment required at recruitment before consent is obtained.

### Fidelity

Completed feedback forms (*N* = 55) were obtained from 10 facilitators (from a total of 12 facilitators) who were involved in delivering the 5 antenatal groups (logs available for 24/30 delivered sessions) and 4 postnatal groups (logs available for 16/24 delivered sessions, see Additional file 5). This was supplemented by 10 observations of antenatal sessions, and 5 observations of postnatal sessions conducted by the parenting programme coordinator.

In the main, only minor content changes were reported from the feedback forms, which included instances of rearranging timings, or changing the order of content. Often this was due to facilitators responding dynamically to the groups’ needs, for example, where participants were already familiar with topic areas.

Overall, observer assessments corroborated the facilitator feedback. Across all observations, the mean fidelity score was 3.3 (out of 4) for topics delivered in the first half of the session, and 3.3 (out of 4) for topics delivered after the break, indicating that facilitators were delivering ‘the majority of elements of the programme’.

### Delivery and costs

Facilitators reported that the delivery of the sessions was straightforward/easy (mean 3.93, SD 0.77, of a 5 point scale where possible answers were 1: complicated/challenging, to 5: straightforward/easy); and that groups engaged well with the materials and concepts (mean 4.21, SD 0.65, 1: not at all engaged to 5: very receptive). Findings from interviews with facilitators corroborated scores.

Each intervention session was delivered by two parenting programme facilitators and took 4.5 h each per session (2.5 h delivery and 1.5 h preparation, 30 min contacting families). This amounted to typical staff costs of £142 per session, assuming a mix of seniority. All rooms were provided free of charge in local Children’s Centres. Three days training time per facilitator were required (typical cost of facilitator time per day, £110).^1^ Materials required in order to run the group (for example, manuals for facilitators, reference books and DVD) cost £425, whilst materials for individual participants (e.g. parenting puzzle book, small gifts such as magnets; gift for child) cost £36 per participant. The total cost of staff time to deliver training was in the region of £1163.^2^ The total cost of the 12 session intervention (including training delivery and attendance, group and individual materials, assuming attendance of 10 participants) was £4312. Additional file 6 contains detailed costings of group and individual materials required to deliver the intervention .

## Discussion

This feasibility study showed that the HAPPY intervention is acceptable, shows promising results for infant obesity prevention, and that a phase III definitive trial to evaluate the intervention is feasible. The study has provided valuable lessons to optimise both a full scale trial and the intervention. The recruitment rate into the feasibility trial was 30 %, which is broadly equivalent to recruitment rates for other similar trials [19–27, 59, 60]. Our findings suggest that any future trial should consider screening approximately three times the number of women required for target recruitment.

The total attrition rate for the trial was 29.2 %, slightly higher than other trials assessing children into early infancy (ranging between 14–25 %); [19, 20] there appeared to be slightly more attrition in the intervention arm (34 %) compared with the control arm (25 %) which remains unexplained. Within the current trial, attempts were made to contact women via telephone at varying times of the day – a minimum of 5 attempts were made. However, we did not use more novel methods of communication such as social media, email or text messaging [60, 61]. In consideration of the definitive trial, we based our sample size calculation on a projected attrition of 25 % as we have learnt key lessons which we feel would improve attrition rates for a full trial. These include the following: inclusion of a home visit for all mothers allocated to the intervention arm by the HAPPY facilitator before the first session to forge a relationship between the woman and the practitioner; improving information and recruitment strategies to ensure that expectant women are fully aware of the commitments of participation; collection of multiple contacts methods at recruitment, including extended family/neighbours and social media identities - and validation of these at recruitment; maintaining regular contact with participants throughout duration of follow-up including repeated contacts and birthday cards for children; design and production of promotional material to continue participant engagement and highlight the value of participation (including a trial website and social media page). A future trial should ensure that a range of communication channels are used to maximise retention.

There was some lack of understanding about the role of the randomisation process or why women had been invited to take part in the study, and this may have impacted on recruitment and/or retention. Discussions surrounding the emotive issues of obesity and the associated risks can be difficult for both women and health professionals [62], but managing expectations of women is vital to ensure commitment to trial measurement schedules.

Some of the measurement tools were found to be burdensome to complete, a problem common to other intervention trials, [23, 63] and interim outcome measures assessed via postal surveys achieved very low response rates indicating their unsuitability for future trials. Assessment of physical activity proved particularly challenging as paper based questionnaires were found to be too burdensome, and participants did not adhere fully to recommended wear times of accelerometers. It is important to strike a balance between collecting valid and reliable data and overly burdening participants, which may lead to missing data, withdrawal or trial attrition.

Our process evaluation suggests high overall acceptability of the intervention to women regularly attending sessions, and to facilitators delivering sessions, which was in line with experiences of other community based parenting programmes [64–66]. Comparison of the attendance rate for HAPPY with other similar trials targeting overweight or obese pregnant women or babies in the first few months of life is challenging as attendance information is inconsistently reported [20–23, 67]. In the current study, 21 out of 26 women who attended at least one antenatal session went on to complete four or more sessions, indicating the importance of first session attendance. One way of encouraging attendance is to ensure a flexible range of dates and locations. Future research could focus on identifying strategies to increase probability of attending the first session, perhaps including some element of flexibility in choice of time or location, and allowing women to meet with the intervention facilitators before the first session, in line with best practice (e.g. [65]).

We found limited information upon which to base our sample size calculation for a full trial, particularly around identifying a minimum clinically important difference, and an appropriate intra-class correlation co-efficient. There is a clear need for more evidence on the effectiveness of *preventive* approaches to reducing obesity amongst infants via high quality randomised controlled trials, and within these trials more robust reporting of relevant statistical information, including effect sizes and intra class correlation co-efficients.

### Limitations

The trial was conducted within a single site, which may not be generalisable to other locations. Within the maternity unit at the Bradford Royal Infirmary, almost 50 % of expectant women are of South Asian origin. This may have implications for generalizability, however it gave us the opportunity to test the intervention in a group traditionally thought of as ‘hard to reach’ [68]. Indeed, we found a higher proportion of South Asian women were recruited compared with White British, indicating that this intervention was found acceptable to a culturally diverse group.

Pragmatic considerations meant that intervention delivery had to be suspended during schools holidays, and screening and recruitment suspended during Ramadan. However, such implementation issues are likely to be common place if the programme is rolled-out nationally.

We were only able to deliver the intervention in English which will have excluded some participants due to language barriers. Following consideration of comments by participants, one pragmatic solution would be to allow participants to bring family members along to intervention sessions to translate, although we recognise this could have consequences on the fidelity of delivered messages. We offered travel reimbursement for parents attending sessions, and we were unable to ascertain whether this impacted on attendance.

The intervention was integrated within the existing FLNP antenatal intervention. Although this makes it easy for children’s service providers in other parts of the country who already have a pool of FLNP trained parenting facilitators to implement the HAPPY intervention, further training will be required for facilitators with no such experience. We recognise limitations of our process evaluation. Due to resource constraints we were unable to transcribe interview scripts, however, interviewers (SA and NS) made detailed field notes and were involved in analysis. We were able to contact only seven women allocated to the intervention group who did not attend sessions, however, responses from these seven women were similar, and in line with recent published research in this area, [69] increasing confidence in our interpretation.

## Conclu***s***ions

The HAPPY intervention is feasible and acceptable to participants who attended and those delivering it. We found the recruitment rate was low at 30 %, although in line with other trials, and study attrition rates were acceptable. However, the majority of women who did not attend the first session failed to attend subsequent intervention sessions. Importantly, this feasibility study has provided an opportunity to develop a clear strategy to enhance attendance (particularly to the first session) prior to the planned definitive trial. On the whole, measurement tools and randomisation posed no major problems for study participants, although there were some issues with the assessment of potential secondary outcomes of physical activity. Participants, facilitators and service providers found the intervention to be useful, and fidelity of implementation was high. Based on these early results, and lessons learned a definitive trial is now justified to assess the effectiveness and cost-effectiveness of the HAPPY intervention at reducing risk of childhood obesity.

## Additional files

Additional file 1:
**Intervention summary – Description of HAPPY intervention.** (PDF 621 kb) Additional file 2:
**Measures – Table of measures assessed at baseline, 6 months and 12 months.** (PDF 297 kb) Additional file 3:
**Additional data tables.** (PDF 275 kb) Additional file 4:
**Details of accelerometry analysis.** (PDF 309 kb) Additional file 5:
**Fidelity analysis: Summary table of participant logs.** (PDF 276 kb) Additional file 6:
**Summary of resources required to deliver the intervention – Table of intervention costs.** (PDF 272 kb)

## Abbreviations

95 % CI: 95 % confidence interval
BMI: body mass index
FLNP: Family Links Nurturing Partnership
HAPPY: Healthy and Active Parenting Programme for early Years
ICC: intra-class correlation co-efficient
IQR: interquartile range
NICE: National Institute for Health and Care Excellence
RCT: randomised controlled trial
SD: standard deviation
SDS: standard deviation score
WHO: World Health Organization

## Footnotes

1 This cost is based on a session being delivered with one family support worker (for illustrative purposes, the salary of a family support worker was costed on NHS agenda for change scales, band 3, point 8, £17,425 per annum gross ( pension and national insurance included) and one senior family support worker (costed at band 5, point 18, £22,903 per annum gross).

2 For illustrative purposes the costing is based on NHS agenda for change scales with two experts delivering the training: programme co-ordinator, band 7, spine 38, £40,097 per annum gross; topic expert, band 8b, spine 39, £61,037 per annum gross).
