# Supplementary material for: The HAPPY (Healthy and Active Parenting Programmme for early Years) feasibility randomised control trial: acceptability and feasibility of an intervention to reduce infant obesity
Source: BMC Public Health. 2016 Mar 1;16:211. doi: 10.1186/s12889-016-2861-z (PMC4774160; doi:10.1186/s12889-016-2861-z)
Supplement: Additional file 1: — Intervention summary – Description of HAPPY intervention. (PDF 621 kb) [file 12889_2016_2861_MOESM1_ESM.pdf]

Additional File 1: Logic model and summary of intervention content

Full details of theoretical basis and intervention development process can be found in Taylor *et al*.,[1]. The intervention comprises 6 ante-natal and 6 post-natal sessions from ~26 weeks gestation until baby is 9 months old. The intervention is delivered in group session format by trained parenting facilitators, with contact by telephone, text and email in between sessions. Figure 1 presents a logic model, and Table 1 presents a summary of intervention content by session, summarising the detailed information presented within Taylor *et al.[1]*

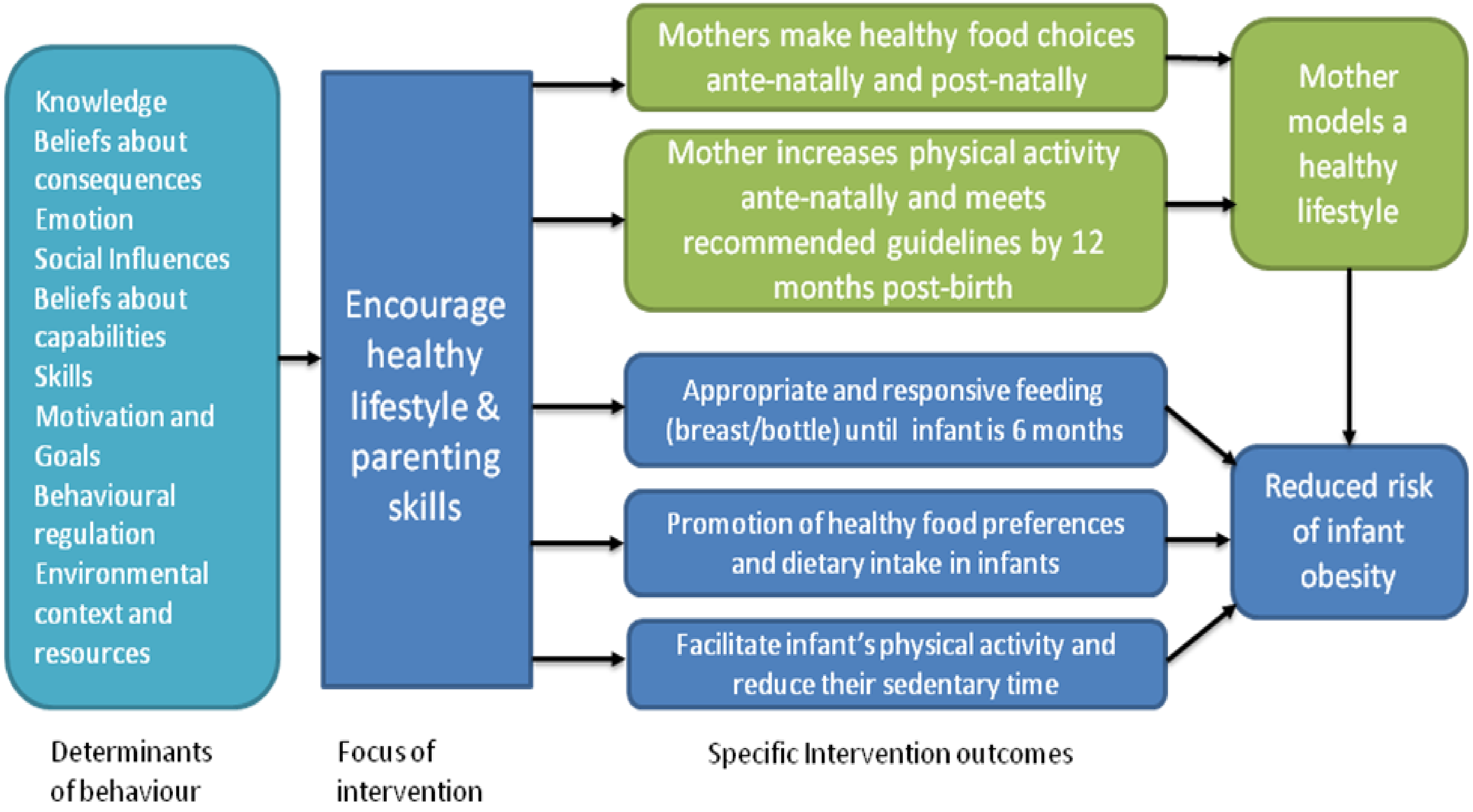

Figure 1 Summary logic model for the HAPPY intervention.

Table 1. Summary of Content

Timing	Session (2.5hrs)	Session content targeting specific risk factors
**Ante natal**	1.Brain, science and bonding	**Parent diet:** Healthy diet in pregnancy; links between what you eat when pregnant, you and your baby; link between maternal and childhood obesity; reflect on own diet and importance of food in the family; **Parent physical activity:** How the baby is developing in the womb and how physical activity can facilitate this; **Breastfeeding:** Identifying and overcoming barriers to breastfeeding
	2.A celebration of birth	**Parent diet:** Importance of eating well for the baby; Dispel myths about weight gain in pregnancy and provide up to date information; Smart Snacks hand-out; food diaries; **Parent physical activity:** Being active during pregnancy; Discussion regarding myths about physical activity in pregnancy
	3.Personal power, self-esteem, and healthy choices about food	**Parent diet:** Address barriers to healthy eating and plan for ways to overcome them; sign post to cooking information points; healthy foods can be convenient and inexpensive; impact of mother eating unhealthy foods; plan alternative cooking methods; **Parent physical activity:** List activities women think they could do, work through easy to difficult; gentle strengthening and conditioning exercises: demonstration by practitioners; worries about physical activity; visualize and positive self-talk; identify when activity can be freely integrated into normal life; **Breastfeeding:** Advantages/ disadvantages of breast and bottle feeding; more you feed, more milk produced; encourage identifying influential family member, clarify likes and dislikes and to talk with the family about these issues to enlist their support; learn more about breastfeeding skills; overcome stigma about breastfeeding in public, stories from other mothers
	4.Boundaries, beliefs and values	**Parent diet:** Additional information about snacks, food treats, swaps; **Parent physical activity:** Back-up plan for physical activity at different times; **Breastfeeding:** Values and beliefs about bringing up children and linking this to breastfeeding
	5.Feelings and how we communicate	**Parent diet:** Food swaps and healthy meals feedback; identify vulnerable points in time and have contingencies for when want unhealthy foods; **Parent physical activity:** Partners review their progress for the collaborative plans made for pa in AN Session 4; discussion about their feelings relating to whether they fulfilled their activity plans
	6.Beyond labour day	**Parent diet:** Identify someone at home/friend to discuss food habits with; **Parent physical activity:** Pelvic floor exercises for immediately after pregnancy if uncomplicated; what activities are okay for after pregnancy; **Breastfeeding:** How to make up bottle correctly; signpost to health professionals; environmental changes: clothes that facilitate feeding; locations nearby; family members can do other things with baby (than formula feed): e.g. bottle feed using expressed milk; **Infant diet:** Planning ahead
**Post natal**	7.A celebration of birth (4wk)	**Parent diet:** Information about what a healthy diet should consist of; food diaries; reflect on own diet and importance of food in the family; **Parent physical activity:** What activities are okay for after pregnancy; gradually introduce more strenuous activity; leaflet with activity examples; physical activity for new mums quiz sheet; practitioner dispels myths; **Breastfeeding:** Reinforce more you feed, more milk produced; identifying someone to obtain support from; responsive feeding; **Infant physical activity:** Guidelines for baby activity; myths about physical activity for babies and infants; practitioner dispels myths; weekly age appropriate activities; encourage structured and unstructured play

Timing	Session (2.5hrs)	Session content targeting specific risk factors
	8.Bringing structure to your life (2mth)	**Parent diet:** Healthy eating for parents vs children; reflect on own eating patterns and provide ideas; balancing diet and avoiding takeaways; **Parent physical activity:** Likes/dislikes about physical activity; activities for after pregnancy; identify barriers; integrating activity into normal life; small changes to family environment; structured activities in the house; the local area; self-talk and visualization; choose a physical activity to learn or can already do; **Breastfeeding:** Identifying barriers; concerns about feeding baby; how to deal with life whilst trying to feed; encourage asking for help to enable breastfeeding to continue and other things to get done; **Infant diet:** Introducing solids: why and when; follow signs of readiness of weaning; **Infant physical activity:** Structured play activities for in the house without expensive toys; introducing games; recognising when baby wants to play; flexible with play-time; making baby-active time a priority
	9.Feeding and feelings (3mth)	**Parent physical activity:** Identifying and addressing barriers; coping planning; the 'Pram Pedometer Challenge'; **Infant diet:** What food and drinks to give; weaning: how to do it - texture and variety, developmental stages, equipment; repeated exposure and coping with food refusal; consequences of an unhealthy diet; facial expressions and tongue thrusting; **Infant physical activity:** Importance of other family members engaging the baby in activity; weekly age appropriate activities for baby and mum; making home modifications to improve child physical activity and/or eating
	10.Families and food (5mth)	**Parent diet:** Feeding a family – what does it take; meal planning; shopping lists; healthy foods can be convenient and inexpensive; reading food labels; impact of mother eating unhealthy foods; **Parent physical activity:** Review of group progress and setting goals for forthcoming weeks; **Infant diet:** Eating together; modelling behaviour and feeding role of family and parents; time issues with food planning and preparation; **Infant physical activity:** Discussion of TV watching; entertaining baby/keeping them safe when doing chores; doing activity as a family; weekly age appropriate activities for baby and mum are demonstrated and practiced; how to baby proof your house for safe physical activity
	11.Making the most of your day (7mth)	**Parent physical activity:** Review of group progress and setting goals for forthcoming weeks; **Infant diet:** Weaning: how's it going? Introducing lumps; **Infant physical activity:** Visualisation and self-talk to build confidence; discussion of barriers and how to overcome them; planning for meal preparation, infant napping, and active play; when can activity can be integrated into normal life; environmental changes; weekly age appropriate activities for baby and mum are demonstrated and practiced; revisit discuss sedentary behaviour and use of TV
	12.Continuing the HAPPY journey (9mth)	**Parent physical activity:** Review of physical activity achievements; reflect on experiences; plan for physical activity future; include follow up monitoring plan by practitioners; announce group winners; **Infant diet:** Keeping motivated to feed family well; **Infant physical activity:** Structured play activities in the house ; involving family and friends with infant play; what local area has to offer; baby-proof your house in a physical activity-friendly way; weekly age appropriate activities; planning for after the parenting programme; joining mum and baby activity groups; signposting and goal setting

1. Taylor NJ, Sahota P, Sargent J, et al. Using intervention mapping to develop a culturally appropriate intervention to prevent childhood obesity: the HAPPY (Healthy and Active Parenting Programme for Early Years) study. The international journal of behavioral nutrition and physical activity 2013;**10**:142 doi: 10.1186/1479-5868-10-142[published Online First: Epub Date]|.
