# Supplementary material for: The HAPPY (Healthy and Active Parenting Programmme for early Years) feasibility randomised control trial: acceptability and feasibility of an intervention to reduce infant obesity
Source: BMC Public Health. 2016 Mar 1;16:211. doi: 10.1186/s12889-016-2861-z (PMC4774160; doi:10.1186/s12889-016-2861-z)
Supplement: Additional file 2: — Measures – Table of measures assessed at baseline, 6 months and 12 months. (PDF 297 kb) [file 12889_2016_2861_MOESM2_ESM.pdf]

Additional file 2: Bespoke measures collected within feasibility study

Study specific items developed for HAPPY feasibility trial

a) Duration of breastfeeding and age of weaning (6 months and 12 months)

1. Was (child's name) ever breast fed? (Cross ONE box only – 6 months only)
Interviewer: Include colostrum in first few days and expressed breast milk

Yes ☐ No ☐

2. Is (child's name) still being breast fed? (Cross ONE box only)

Yes ☐ No ☐

Interviewer If YES go to question 4

3. How old was (child's name) when he/she completely stopped being breastfed?
Interviewer: Include expressed breast milk? _______

☐ Still having breast milk

4. How old was (child's name) when he/she was first given baby milk formula to drink?
Interviewer: SMA, Cow & Gate, Formula Soya milk, Follow-on formula milk etc? ______

☐ Still not had formula milk

5. How old was (child's name) when he/she was given a sweetened drink? This does not include breast or formula milk or other low sugar or non-sweetened drinks. _________

☐ Still not had anything else to drink

6. How old was (child's name) when he/she was given a non- sweetened drink such as tap or mineral water, unsweetened herbal drink, unsweetened fruit juice, diet drinks low in sugar including diet cola or diet squash and unsweetened tea? ________

☐ Still not had anything else to drink

7. How old was (child's name) when he/she was given savoury solids to eat such as baby foods in a jar, packet, tin or homemade? (e.g. baby rice, pre-prepared baby foods, pureed vegetables, finger foods or rice, lentils/dahl etc.) ______

☐ Still not had any savoury solids

8. How old was (child's name) when he/she was given sweet solids to eat? Such as sweet baby foods in a jar, packet, tin or homemade (e.g. egg custard, rice pudding, sweetened rusks, biscuits, cake etc.) _________

☐ Still not had any solids

9. At what age was (child's name) first introduced to a feeding cup?
(A feeding cup is a cup or beaker without a teat used to feed a child) *(6 month questionnaire only)*

Age in Months ____ Never ☐

b) Additional infant feeding questions in 12 months questionnaire

1. Does *(child's name)* drink from… (Please tick as appropriate)

A bottle with a teat?	☐	A bottle with a spout?	☐
A beaker or feeding cup?	☐	none of these	☐

2. At what age did you introduce a beaker or cup? _______

☐ Have not used a beaker or feeding cup

3. At home, which of the following do you normally give *(child's name)*…
(Tick all that apply)

Always give homemade food ☐
Always give commercial (tins, jars, packets) food ☐
Give both homemade and commercial food. ☐

C) Infant physical activity: tummy time questions (6 months)

1a. How often does (child's name) spend time on his/her tummy whilst he/she is awake?

- ☐ Everyday
- ☐ 4- 6 days a week
- ☐ 2-3 days a week
- ☐ One day a week
- ☐ Occasionally
- ☐ Never

1b. and for how long each day? _____ minutes per day

[Prompt: how much time in the morning, in the afternoon, in the evening?]

2. At what age did (child's name) start spending time on his/her tummy whilst he/she was awake? _________

[Prompt: less than 3 months old, 3-6 months old, 6-9 months old? Encourage the mother to give exact month/weeks of age]

D) Infant sedentary time (12 months only)

1a. How often does (child’s name) spend time in front of the television when he/she is awake?

- ☐ Everyday
- ☐ 4- 6 days a week
- ☐ 2-3 days a week
- ☐ One day a week
- ☐ Occasionally
- ☐ Never

1b. and for how long each day? ______ minutes per day

[Prompt: how much time in the morning, in the afternoon, in the evening?]

E) Infant sitting questions (6 months and 12 months)

1. How often when (child’s name) is awake is he/she inactive for longer than 60 minutes at a time?

[prompt: in a pushchair, in a highchair outside of meal times, in a car seat, in their cot?]

- ☐ Everyday
- ☐ 4- 6 days a week
- ☐ 2-3 days a week
- ☐ Once day a week
- ☐ Occasionally
- ☐ Never

F) Infant sitting (12 months only)

1a. How often does *(child’s name)* spend time watching television (or DVDs) when he/she is awake?

- ☐ Everyday
- ☐ 4- 6 days a week
- ☐ 2-3 days a week
- ☐ One day a week
- ☐ Occasionally
- ☐ Never

1b. and for how long each day? ____ minutes per day

G) Modified cost questionnaire (Richardson et al, 2008)

Section D- Use Of Hospital Services

1. Have you or (child's name) had any overnight stays in hospital **since (child's name) was born?**

☐ Yes ☐ No

If **YES**, please give details of the number of nights you were in hospital (approximate if not sure). Please give details of each stay on a different line.

Department/ Speciality	Number of Nights	
	You	Child's name

2a. Have you or your child had any outpatient appointments **since your child was born?**

☐ Yes ☐ No

2b. If **YES**, please give details of the number of visits you have made to each department/speciality. If in more than one department, please list the number in each (e.g. 6 appointments, 4 in Maternity, 2 in Diabetes clinic, 1 in Paediatrics).

Department/Speciality	Number of appointments kept		Number of missed or cancelled appointments	
	You	Child's name	You	Child's name

3a. Have you or your child had treatment at an Accident and Emergency department **since (child's name) was born?**

☐ Yes ☐ No

3b. If **YES**, please give number of visits.

Number of visits	
You	Child's name

Section E- Use Of Services outside the hospital

Please estimate the total number of times you have used each of the services below, **since the birth of (child's name)**. *Please answer for both yourself and (child's name). (Please enter '0' if a particular service was not used):*

	Use	n/a	don't know	does not wish to answer
1. General Practitioner (at the surgery)	☐	☐	☐	☐
2. General Practitioner (at your home)	☐	☐	☐	☐
3. Practice Nurse (at GP surgery)	☐	☐	☐	☐
4. Midwife	☐	☐	☐	☐
5. Midwifery Support Worker (e.g. breast feeding)	☐	☐	☐	☐
6. Health Visitor (only number of Home Visits)	☐	☐	☐	☐
7. Health Visitor Support Worker (Only number of Home Visits)	☐	☐	☐	☐
8. Physiotherapy	☐	☐	☐	☐
9. Child Health Clinics (at GP surgery or Health Centre)	☐	☐	☐	☐
10. Children's Centre	☐	☐	☐	☐
11. Gym	☐	☐	☐	☐
12. Other (please specify)	☐	☐	☐	☐

____

13. Have you had any out of pocket health related expenses (e.g. had to pay for any over the counter drugs) over the last 6 months?

☐ ☐
