# Supplementary material for: The HAPPY (Healthy and Active Parenting Programmme for early Years) feasibility randomised control trial: acceptability and feasibility of an intervention to reduce infant obesity
Source: BMC Public Health. 2016 Mar 1;16:211. doi: 10.1186/s12889-016-2861-z (PMC4774160; doi:10.1186/s12889-016-2861-z)
Supplement: Additional file 3: — Additional data tables. (PDF 275 kb) [file 12889_2016_2861_MOESM3_ESM.pdf]

Additional file 3 Methodology for calculating the number of days of accelerometry required to reliably estimate mothers' and infants' physical activity

The wear time criteria of at least 10 hours on a minimum of 3 days has commonly been applied in order to have enough wear time to reliably estimate habitual physical activity levels in adults [1, 2]. However this wear time criteria is not sample-specific, therefore participants with unreliable/reliable physical activity data may be excluded or included. In very young children there are no established wear-time criteria. In order to maximise the number of participants with reliable data, the Spearman-Brown prophecy formula [3] and methods outlined by Hinkley and colleagues [4] (investigating weekday/weekend difference and reactivity) were applied to mothers and infants accelerometer data to determine minimum wear time (daily-wear hours and number of wear days) required to achieve a reliable estimate of habitual physical activity (intraclass correlation coefficient (ICC) value ≥ 0.8 [5])[1].

<u>Results</u>

Table 4.1 shows that for mothers to reach an ICC of 0.8, seven to nine hours wear is required on three days. If six or less hours of wear were to be selected, four or five days wear would be required. Thus the minimum wear time of 7 hours on three days is enough to reliably estimate habitual physical activity levels (moderate to vigorous physical activity (MVPA)) of mothers. A t-test and one-way within-subject ANOVA found that weekday and weekend day difference and reactivity (difference across days) and did not exist for mother's data, therefore any day of data could be included in the analysis.

Table 4.2 shows that few infants wore the accelerometers for more than seven hours a day and thus the reliability scores for these hours are likely spurious; thus only wear of six or less hours was considered in the reliability assessment. For infants to reach an ICC of 0.8, five to six hours wear is required on two days.

---

[1] Spearman–Brown prophecy:

Estimated days = [Desired *r* * (1 − SingledayICC))/(SingledayICC * (1 − Desired *r*)]

For example:

Estimated days = [0.8 * (1 − SingledayICC))/SingledayICC * (1 − 0.8)]

If four or less hours of wear were to be selected, three or four days would be required. Thus a minimum of five hours on two days would be enough to reliably estimate habitual physical activity levels. A t-test and one-way within-subject ANOVA found that weekday and weekend day difference and reactivity (difference across days) and did not exist for infant's data, therefore any day of data could be included in the analysis.

Table 4.1 : Mother single day ICC and estimated number of needs required to reach reliability.

No. Hours of any 3 random days	No. Participants	Outcome* (%)	Single Day ICC	95% CL	Minimum days needed for 0.8 reliability (Spearman-Brown) †	Minimum days needed for 0.7 reliability (Spearman-Brown) †
9 hours	23	MVPA	0.65	0.52-0.65	2.15	1.26
8 hours	26	MVPA	0.63	0.45-0.75	2.35	1.37
7 hours	28	MVPA	0.62	0.61-0.72	2.45	1.43
6 hours	29	MVPA	0.47	0.37-0.57	4.51	2.63
5 hours	31	MVPA	0.51	0.39-0.63	3.84	2.24
4 hours	35	MVPA	0.52	0.41-0.61	3.69	2.15
3 hours	40	MVPA	0.55	0.32-0.71	3.27	1.91

*% Moderate to Vigorous Physical Activity (MVPA). This is the chosen physical activity outcome for mothers.

† The number of days required for reliability were rounded upwards to the nearest day, so for example 2.1 (days) and 2.7 (days) would be rounded up to 3 (days).

Table 4.2 Infant single day ICC and estimated number of needs required to reach reliability

No. Hours of any 3 random days	No. Participants	Outcome*	Single Day ICC	95% CL	Minimum days needed for 0.8 reliability (Spearman-Brown) †	Minimum days needed for 0.7 reliability (Spearman-Brown) †
9 hours	6	CPM	0.66	0.55 - 0.77	2.06	1.20
8 hours	11	CPM	0.67	0.53 - 0.81	1.97	1.15
7 hours	14	CPM	0.55	0.41 - 0.68	3.27	1.91
6 hours	21	CPM	0.77	0.65 - 0.89	1.19	0.70
5 hours	25	CPM	0.78	0.63 - 0.93	1.13	0.66

4 hours	28	CPM	0.65	0.40 - 0.90	2.15	1.26
3 hours	32	CPM	0.57	0.35 - 0.79	3.02	1.76

*CPM, Counts per minute. This is the chosen physical activity outcome for infants.

† The number of days required for reliability were rounded upwards to the nearest day, so for example 2.1 (days) and 2.7 (days) would be rounded up to 3 (days).

1. Matthews CE, Chen KY, Freedson PS, et al. Amount of time spent in sedentary behaviors in the united states, 2003-2004. American Journal of Epidemiology 2008;**167**(7):875-81 doi: 10.1093/aje/kwm390[published Online First: Epub Date]|.
2. Troiano RP, Berrigan D, Dodd KW, Masse LC, Tilert T, McDowell M. Physical activity in the United States measured by accelerometer. Medicine and Science in Sports and Exercise 2008;**40**(1):181-88 doi: 10.1249/mss.0b013e31815a51b3[published Online First: Epub Date]|.
3. Baumgartner TA. Applicability of Spearrman-Brown Prophecy formula when applied to physical performance tests. Research Quarterly 1968;**39**(4):847-56
4. Hinkley T, O'Connell E, Okely AD, Crawford D, Hesketh K, Salmon J. Assessing Volume of Accelerometry Data for Reliability in Preschool Children. Medicine and Science in Sports and Exercise 2012;**44**(12):2436-41 doi: 10.1249/MSS.0b013e3182661478[published Online First: Epub Date]|.
5. Stanley JC. *Epidemiology*. Washington DC: Thorndike RL, 1971.
