# Supplementary material for: The HAPPY (Healthy and Active Parenting Programmme for early Years) feasibility randomised control trial: acceptability and feasibility of an intervention to reduce infant obesity
Source: BMC Public Health. 2016 Mar 1;16:211. doi: 10.1186/s12889-016-2861-z (PMC4774160; doi:10.1186/s12889-016-2861-z)
Supplement: Additional file 4: — Details of accelerometry analysis. (PDF 309 kb) [file 12889_2016_2861_MOESM4_ESM.pdf]

Additional file 4: Additional data tables

Table 3.1 Logistic regression to predict recruitment into HAPPY trial (N=189 with complete data)

**Variable**	Odds Ratio (95% Confidence interval
Maternal age at screening	0.99 (0.93, 1.06)
Gestational age at screening	0.94 (0.86, 1.02)
Parity	1.01 (0.81, 1.27)
Body Mass Index at booking	1.00 (0.94, 1.07)
Ethnicity	
South Asian origin (reference)	1
White British	***0.48 (0.22, 0.85)****
Other ethnicity	1.21 (0.53, 2.79)

Note: * p<.05

Table 3.2 Multivariable regression to predict attendance at intervention sessions (intervention group only, N=53 with complete data)

**Variable**	β (95% confidence interval)
Maternal age at screening	0.16 (-0.02-0.35)
Gestational age at screening	-0.01 (-0.21-0.19)
Parity	-0.25 (-0.92 – 0.41)
Body Mass Index at booking	-0.04 (-0.21-0.124)
Ethnicity	
South Asian origin (reference)	1
White British	0.36 (-1.41-2.12)
Other ethnicity	0.89 (-1.22 – 2.99)

Note: no predictors significant

Table 3.3 Six-month follow-up data

**Variable**	**All N=83**	**Intervention N=42**	**Control N=41**
Age of child at time of questionnaire (months)	6.2 (0.6)	6.3 (0.6)	6.1 (0.5)
Child's length available	15 (18.1)	7 (16.6%)	8 (19.5)
Child's weight available	57 (68.7)	31 (73.8)	26 (63.4)
**Child diet**			
Child ever breastfed	58 (69.9)	31 (73.8)	27 (65.9)
Child still being breastfed	23 (27.7)	14 (33.3)	9 (21.9)
Duration of breastfeeding (weeks – of those who reported cessation of breastfeeding) $	4.0 (1.0, 12.9)	3.0 (2.0, 8.6)	4.7 (0.1, 14.9)
Age given formula (weeks) $<br>Not had formula	0.3 (0.1, 4.0)<br>10 (12.1)	0.4 (0.1, 4.2)<br>6 (14.3)	0.1 (0.1, 2.0)<br>4 (9.8)
Age given sweetened drinks (weeks) $<br>Not had sweetened drinks	21.5 (17.2, 21.5)<br>58 (69.9)	21.5 (17.2, 25.8)<br>28 (66.7)	21.5 (17.2, 21.5)<br>30 (73.2)
Age given solids (weeks) $<br>Not had solids	21.5 (17.2, 25.8)<br>6 (7.2)	21.5 (17.2, 25.8)<br>4 (9.5)	21.5 (17.2, 25.8)<br>2 (4.9)

Variable	All<br>N=83	Intervention<br>N=42	Control<br>N=41
Age introduced to a feeding cup (weeks)<br>Not used feeding cup	5.0 (0.9)<br><br>38 (45.8)	5.0 (1.0)<br><br>17 (40.5)	5.0 (0.8)<br><br>21 (51.2)
**Maternal physical activity**			
Hours spent sitting each week day[$]<br>Missing<br><br>Hours spent sitting each weekend day[$]<br>Missing	3.5 (2.3, 5.3)<br>1 (1.2)<br><br>3.6 (2.3, 6.0)<br><br>1 (1.2)	3.7 (2.2, 5.6)<br>1 (2.4)<br><br>3.7 (2.3, 6.0)<br><br>1 (2.4)	3.3 (2.3, 5.0)<br>0<br><br>3.5 (2.3, 6.0)<br><br>0
**Child physical activity**			
Ever spent time on tummy whilst awake<br>Yes<br>Missing	 <br>79 (95.2)<br>1 (1.2)	 <br>40 (95.2)<br>0	 <br>39 (95.1)<br>1 (2.4)
Age started spending time on tummy (weeks) [$]	17.2 (10.0, 21.5)	17.2 (8.6, 21.5)	17.2 (12.9, 21.5)
Minutes per day spent on tummy[$]	20.0 (5.0, 70.0)	30.0 (6.1, 90.0)	10.7 (3.6, 60.0)
Minutes per day spent in front of TV[$]	17.5 (0.7, 40.0)	30.0 (1.1, 60.0)	10.4 (0.5, 30.0)
**Child development**			
Returned ages and stages questionnaire<br>Yes	 <br>32 (38.5)	 <br>15 (35.7)	 <br>17 (41.4)
**Health Economic Measures**			
**Use of Hospital Services**			
Had overnight stay in hospital<br>Yes<br>Missing<br><br>Had outpatient appointment<br>Yes<br>Missing<br><br>Had A&E Attendance<br>Yes<br>Missing	 18 (21.6)<br>1 (1.2)<br><br><br>22 (26.5)<br>1 (1.2)<br><br><br>23 (27.7)<br>1 (1.2)	 12 (28.6)<br><br><br><br>13 (31.0)<br><br><br><br>14 (33.3) 	 6 (14.6)<br>1 (2.4)<br><br><br>9 (21.9)<br>1 (2.4)<br><br><br>9 (21.9)<br>1 (2.4)
**Use of services outside the hospital**			
General practitioner (at the surgery)<br>Used<br>Missing	 79 (95.2)<br>1 (1.2)	 41 (97.6)<br>0	 38 (92.7)<br>1 (2.4)
General practitioner (at your home)<br>Used<br>Missing	 1 (1.2)<br>1 (1.2)	 0	 1 (2.4)<br>1 (2.4)

Variable	All<br>N=83	Intervention<br>N=42	Control<br>N=41
Practice nurse (at GP surgery)<br>Used<br>Missing	 67 (80.7)<br>1 (1.2)	 33 (78.6)<br>0	 34 (82.9)<br>1 (2.4)
Midwife<br>Used<br>Missing	 67 (80.7)<br>1 (1.2)	 33 (78.6)<br>0	 34 (82.9)<br>1 (2.4)
Midwife support worker<br>Used<br>Missing	 8 (9.6)<br>1 (1.2)	 6 (14.3)<br>0	 2 (4.9)<br>1 (2.4)
Health visitor (home visits)<br>Used<br>Missing	 79 (95.2)<br>1 (1.2)	 40 (95.2)<br>0	 39 (95.1)<br>1 (2.4)
Health visitor support worker (home visits)<br>Used<br>Missing	 10 (12.1)<br>1 (1.2)	 34 (81.0)<br>0	 36 (87.8)<br>1 (2.4)
Physiotherapy<br>Used<br>Missing	 4 (4.8)<br>0 (1.2)	 2 (4.8)<br>0	 2 (4.9)<br>1 (2.4)
Child health clinics<br>Used<br>Missing	 27 (32.5)<br>1 (1.2)	 14 (33.3)<br>0	 13 (31.7)<br>1 (2.4)
Children's Centre<br>Used<br>Missing	 29 (34.9)<br>1 (1.2)	 13 (31.0)<br>0	 16 (39.0)<br>1 (2.4)
Gym<br>Used<br>Missing	 6 (7.2)<br>1 (1.2)	 2 (4.8)<br>0	 4 (9.8)<br>1 (2.4)
Used other services<br>Used<br>Missing	 3 (3.6)<br>1 (1.2)	 1 (2.4)<br>0	 2 (4.9)<br>1 (2.4)
Have you had any out of pockets health related expenses over the last 6 months<br>Yes<br>Missing	 35 (42.2)<br>1 (1.2)	 21 (50.0)<br>0	 14 (34.2)<br>1 (2.4)

NB Data are mean (SD), n (%) or median (IQR) unless otherwise stated. The latter is denoted with $

Table 3.4 Twelve month follow-up data

Variable	All (N=85)	Intervention (N=39)	Control (N=46)
Mothers' BMI $<br>Missing	29.0 (26.0, 32.0)<br>3 (3.5)	28.0 (26.0, 30.0)<br>1 (2.6)	29.0 (26.0, 33.0)<br>2 (4.3)
**Maternal diet**			
Daily fruit and vegetable intake $	4.0 (2.0, 7.0)	5.0 (2.0, 9.0)	4.0 (2.0, 7.0)
Foods present in the home			
Fruit	85 (100.0)	39 (100.0)	46 (100.0)
Vegetables	85 (100.0)	39 (100.0)	46 (100.0)
Snacks	83 (97.7)	39 (100.0)	44 (95.7)
Fizzy drinks (exc. Diet drinks)	75 (88.2)	36 (92.3)	39 (84.8)
Quantity of foods present in the home (portions)			
Fruit $	7.0 (5.0, 9.0)	7.0 (5.0, 9.0)	7.0 (6.0, 8.0)
Vegetables$	8.0 (6.0, 9.0)	8.0 (6.0, 10.0)	8.0 (7.0, 9.0)
Snacks $	5.0 (4.0, 6.0)	5.0 (3.0, 6.0)	4.0 (4.0, 5.0)
Fizzy drinks (exc. Diet drinks) $	2.0 (1.0, 2.0)	2.0 (1.0, 2.0)	2.0 (1.0, 2.0)
Food frequency questionnaire: completed	80	36	44
Formula (per day)	0.0 (0.0, 2.0)	0.0 (0.0, 2.0)	0.2 (0.0, 2.9)
Commercial savoury baby meals (per week)	0.0 (0.0, 2.5)	0.0 (0.0, 3.0)	0.0 (0.0, 2.0)
Commercial sweet baby meals (per week)	0.0 (0.0, 0.0)	0.0 (0.0, 2.0)	0.0 (0.0, 0.0)
Chips, roast and potato shapes (per week)	1.0 (0.0, 2.0)	1.0 (0.0, 2.0)	1.0 (0.0, 2.0)
Processed meat products (per week)	0.3 (0.0, 2.0)	0.0 (0.0, 2.0)	0.5 (0.0, 2.5)
Vegetables (per day)	1.6 (1.0, 2.5)	1.6 (1.1, 2.7)	1.6 (0.8, 2.4)
Fruit (per day)	1.8 (1.0, 2.9)	1.7 (1.0, 2.2)	1.9 (1.0, 3.1)
Sweet snacks (per day)	0.6 (0.1, 1.2)	0.3 (0.1, 1.3)	0.6 (0.2, 1.1)
Savoury snacks (per week)	0.0 (0.0, 2.0)	0.0 (0.0, 1.5)	0.0 (0.0, 2.0)
Sugar sweetened drinks (per week)	2.0 (0.0, 7.5)	1.5 (0.0, 11.0)	2.0 (0.0, 7.0)
Pure fruit juice (per week)	0.5 (0.0, 7.0)	0.3 (0.0, 5.0)	0.7 (0.0, 7.0)
Low sugar drinks (per week)	0.0 (0.0, 0.0)	0.0 (0.0, 0.0)	0.0 (0.0, 0.0)
Water (per day)	3.0 (2.0, 4.0)	3.5 (2.0, 5.0)	3.0 (2.0, 4.0)
**Maternal physical activity**			
Hours spent sitting each week day$	5.4 (3.3, 7.3)	6.0 (3.8, 8.0)	4.3 (3.0, 7.0)
Hours spent sitting each weekend day$	5.0 (3.0, 7.8)	4.8 (3.0, 8.5)	5.0 (3.0, 7.5)
**Accelerometry**			
**Mothers**			
Number accepting an accelerometer	78	34	44
Number who wore belt for at least 7 hours on at least 3 days	28 (35.9)	10 (29.4)	18 (40.9)
Median moderate – vigorous physical activity minutes per day (of those who wore belt)	13.8 (9.3, 33.0)	20.3 (9.6, 38.9)	13.6 (9.0, 23.7)
Median sedentary minutes per day (of those who wore belt)	356. (297.5, 404.2)	338.3 (258.2, 386.0)	370.7 (303.3, 408.7)
**Children**			

Variable	All (N=85)	Intervention (N=39)	Control (N=46)
Number consenting to child wear an accelerometer[1]	73	31	41
Number children who wore belt for at least 5 hours on at least 3 days	33 (45.8)	10 (32.2)	23 (56.1)
Median counts per minute (of those who wore belt)	230.9 (176.2, 385.6)	183.7 (134.7, 198.8)	284.8 (196.9, 406.8)
**Child diet**			
Child ever breastfed	60 (70.6)	28 (71.8)	32 (69.6)
Duration of breastfeeding (weeks) $	8.6 (4.0, 17.2)	7.8 (4.0, 17.2)	8.6 (3.0, 17.2)
Age introduced to solid food (weeks)	24.3 (5.0)	24.7 (5.5)	24.0 (4.6)
**Child development**			
'Normal' motor development (ASQ)<br>Missing	70 (84.0)<br>2	31 (83.8)<br>2	39 (84.8)<br>0
**Parenting practices**			
Low parental self-efficacy (%)	17 (20.0)	10 (25.6)	7 (15.2)
Low parental warmth (%)	21 (24.7)	8 (20.5)	13 (28.3)
Hostile parenting (%)	17 (20.0)	7 (18.0)	10 (21.7)
**Feeding styles**			
Concern about the child under eating or becoming underweight$12	1.3 (0.5, 2.3)	1.8 (1.0, 2.5)	1.0 (0.3, 2.0)
Concern about infant's hunger$12	0.3 (0.0, 1.0)	0.3 (0.0, 1.0)	0.3 (0.0, 1.0)
Awareness of infant's cues$13	3.8 (3.3, 4.0)	3.8 (3.0, 4.0)	3.8 (3.3, 4.0)
Concern about the infant becoming overweight or overeating$12	0.0 (0.0, 0.7)	0.3 (0.0, 1.0)	0.0 (0.0, 0.7)
Feeding the infant on schedule$14	2.5 (2.0, 2.5)	2.0 (1.5, 2.5)	2.5 (2.0, 3.0)
Using food to calm the infant$14	1.5 (0.5, 2.0)	1.5 (1.0, 2.5)	1.0 (0.5, 2.0)
Social interaction during feeding$14	2.5 (2.0, 3.0)	3.0 (2.0, 3.0)	2.5 (2.0, 3.5)
**Health Economic Measures**			
**Use of Hospital Services**			
Had overnight stay in hospital<br>Yes<br>Missing<br><br>Had outpatient appointment<br>Yes<br>Missing<br><br>Had A&E Attendance<br>Yes<br>Missing	 7 (8.2)<br>0<br><br><br>24 (28.2)<br>0<br><br><br>17 (20.0)<br>0	 3 (7.7)<br>0<br><br><br>10 (25.6)<br>0<br><br><br>6 (15.4)<br>0	 4 (8.6)<br>0<br><br><br>14 (30.4)<br>0<br><br><br>11 (23.9)
**Use of services outside the hospital**			
General practitioner (at the surgery)<br>Used<br>Missing	 76 (89.4)<br>0	 35 (89.7)<br>0	 41 (89.1)<br>0

[1] Due to a clerical error, the accelerometry data of one infant could not be linked to their participant ID, thus we were unable to ascertain whether they were part of the intervention or control group

Variable	All<br>(N=85)	Intervention<br>(N=39)	Control<br>(N=46)
General practitioner (at your home)<br>Used<br>Missing	 1 (2.6)<br>0	 1 (2.6)<br>0	 0<br>0
Practice nurse (at GP surgery)<br>Used<br>Missing	 55 (64.7)<br>0	 22 (56.4)<br>0	 33 (71.7)<br>0
Midwife<br>Used<br>Missing	 11 (12.9)<br>0	 6 (15.4)<br>0	 5 (10.9)<br>0
Midwife support worker<br>Used<br>Missing	 2 (2.4)<br>0	 0<br>0	 2 (4.4)<br>0
Health visitor (home visits)<br>Used<br>Missing	 36 (42.4)<br>0	 17 (43.6)<br>0	 19 (41.3)<br>0
Health visitor support worker (home visits)<br>Used<br>Missing	 <br>2 (2.6%)<br>0	 <br>2 (5.1)<br>0	 <br>0<br>0
Physiotherapy<br>Used<br>Missing	 8 (9.4)<br>0	 3 (7.7)<br>0	 5 (10.9)<br>0
Child health clinics<br>Used<br>Missing	 18 (21.2)<br>0	 7 (18.0)<br>0	 11 (23.9)<br>0
Children's Centre<br>Used<br>Missing	 30 (35.3)<br>0	 10 (25.6)<br>0	 20 (43.5)<br>0
Gym<br>Used<br>Missing	 10 (11.8)<br>0	 5 (12.8)<br>0	 5 (10.9)<br>0
Used other services<br>Used<br>Missing	 1 (1.2)<br>0	 1 (2.6)<br>0	 0<br>0
Have you had any out of pocket health related expenses over the last 6 months<br>Yes<br>Missing	 <br>18 (21.2)<br>0	 <br>12 (30.8)<br>0	 <br>6 (13.0)<br>0
EQ-5D Measure<br>Mobility<br>No problems in walking about<br>Some problems walking about<br>Confined to bed<br>Missing	 <br>75 (88.2)<br>9 (10.6)<br>1 (1.2)<br>0	 <br>34 (87.2)<br>4 (10.3)<br>1 (2.6)<br>0	 <br>41 (89.1)<br>5 (10.9)<br>0<br>0
Self care<br>No problems with self care<br>Some problems with self care<br>Unable to wash or dress my self	 82 (96.5)<br>3 (3.5)<br>0	 37<br>2<br>0	 45<br>1<br>0

**Variable**	**All (N=85)**	**Intervention (N=39)**	**Control (N=46)**
Missing	0	0	0
Usual activities			
No problems performing usual activities	76 (89.4)	33 (84.6)	43 (93.5)
Some problems performing using activities	9 (10.6)	6 (15.4)	3 (6.5)
Unable to perform usual activities			
Missing	0	0	0
	0	0	0
Pain / Discomfort			
No pain or discomfort	58 (68.2)	24 (61.5)	34 (73.9)
Moderate pain or discomfort	27 (31.8)	15 (38.5)	12 (26.1)
Extreme pain or discomfort	0	0	0
Missing	0	0	0
Anxiety / Depression			
Not anxious or depressed	78 (91.7)	43 (87.1)	44 (95.7)
Moderately anxious or depressed	5 (5.9)	4 (10.3)	1 (2.2)
Extremely anxious or depressed	2 (2.4)	1 (2.6)	1 (2.2)
Missing	0	0	0
How is your health today? (range worst imaginable health state: 0- Best imaginable health state: 100)	67.5 (18.6)	61.4 (17.8)	72.6 (17.9)

Data are mean (SD), n (%) or median (IQR) unless otherwise stated. The latter is denoted with [$]. [1] Maximum score=4; [2] Higher=more concern; [3] Higher=more aware; [4] Higher=more likely
