# Supplementary material for: The HAPPY (Healthy and Active Parenting Programmme for early Years) feasibility randomised control trial: acceptability and feasibility of an intervention to reduce infant obesity
Source: BMC Public Health. 2016 Mar 1;16:211. doi: 10.1186/s12889-016-2861-z (PMC4774160; doi:10.1186/s12889-016-2861-z)
Supplement: Additional file 5: — Fidelity analysis: Summary table of participant logs. (PDF 276 kb) [file 12889_2016_2861_MOESM5_ESM.pdf]

Additional file 5: Intervention fidelity by session (Facilitator logs)

Session	Number of logs received	Ease of delivery: Mean (SD)[a]	Participants engaged? Mean (SD)[b]	Any change to content?	Comments on timings	Notes about materials
AN1	9	3.83 (0.35)	3.83 (0.61)	2/5 groups reported minor changes to content:<br>**Group A**: Modified timings to give increased time on one topic and to decrease another<br>**Group D**: Omitted one section (attunement / name game); rearranged order (introduced nurturing programme before ice breaker	4/5 groups reported feeling rushed due to starting late or having to complete paperwork: e.g., "Very rushed due to late start and paperwork at the beginning."	No problems with materials were reported
AN2	7	4.57 (0.53)	4.29 (0.49)	2/5 groups reported minor changes to content<br>**Group A**: Changed order to aid 'flow'<br>**Group D**: included section omitted from previous week; modified one section 'me & you' game<br>**Group E**: *Put the flipcharts for changes in me + you in a different order*	4/4 groups reported timings worked well	2/5 groups reported some problems with too many hand-outs<br>e.g. "It was fine but too many hand-outs - so parents were confused"<br><br>1 group reported confusion on cultural understanding.<br>"… The only thing complicated was cultural understanding: people's different cultures and confusion on thoughts."
AN3	7	3.07 (1.02)	3.86 (0.69)	3/4 groups reported changes:<br>**Group A**: edited content for key messages (vitamin D, breastfeeding)<br>**Group B**: omitted 2 sections 'touch' and 'concerns about	4/4 groups reported this session was rushed.<br>E.g.<br>"Too much information for this session."<br>"The session content was too	1/4 groups reported some problems with material:<br>e.g. "I thought some of the material repeated itself."<br><br>Otherwise materials were fine:

Session	Number of logs received	Ease of delivery: Mean (SD)[a]	Participants engaged? Mean (SD)[b]	Any change to content?	Comments on timings	Notes about materials
				infant feeding'<br>**Group D**: omitted sections: Changing Places, Relaxation, Healthy Meal Challenge, Touch, Self-Esteem and Physical Activity	long - timings were an issue as discussions were cut short as we needed to cover other things"	e.g.<br>"Easy to follow."<br>"material was fine"
AN4	4	4.50 (0.58)	4.00 (0)	2/3 groups reported minor content changes:<br>**Group A:** Edited some sections for key content<br>**Group D:** Discussion shortened (nurturing ourselves, nurturing physical activity)	2/3 groups reported no problems with timings<br>e.g.,<br>"Worked very well, more straightforward activities and was easier to deliver contents and keep to the time."<br>1 group reported a minor problem<br>"Getting Started was difficult - late arriving and lots of chat…"	1/3 groups reported problems with hand-outs<br>"Hand-outs may have too much information. Learners do not seem to be reading or giving positive information on them."
AN5	5	3.75 (0.50)	4.50 (0.71)	3/5 groups reported minor content changes 2 groups omitted one task (the 'I' task: Groups B & D)<br>Group A: reported minor changes to one activity (handling stress)	5/5 groups felt session was rushed<br>e.g. "Timings were an issue as the session was yet another long one - so it was difficult to get all the information across"	2/5 groups reported no problems with material<br>e.g., "fine"<br>2/5 groups reported that there was "too much material"
AN6	5	4.00 (0.71)	4.50 (0.58)	1/ 4 groups reported minor changes to content:<br>Group D: omitted 'common complaints', edited 'empathy' section.	No problems with timings were reported<br>e.g.,<br>"Really well - got through everything on time."	2/4 groups reported problems with materials<br>e.g.<br>"Quiz was complicated: could change the questions, relate it to the programme."

Session	Number of logs received	Ease of delivery: Mean (SD)[a]	Participants engaged? Mean (SD)[b]	Any change to content?	Comments on timings	Notes about materials
						“Material could have been shortened, more condensed.”
PN1	4	4.25 (0.96)	4.25 (0.50)	One group (D/E) omitted content due to late arrival of mums	2/3 Groups reported no problems with timings e.g. “worked well and fit it all in”<br>1 Group started late due to adverse weather conditions so struggled to fit all content in.	No groups reported problems with materials
PN2	5	3.60 (0.55)	4.00 (0.82)	2/4 groups reported minor changes to content<br>Group B reported omitted one section (unnamed)<br>Group A: omitted DVD clip, but and shortened discussion on ‘boundaries’	1/4 Groups reported no problems with timings<br>3/4 Groups reported problems with timings due to late arrivals E.G. “Took a long time to get started due to late arrivals etc.”<br>Or amount of content<br>e.g. “a lot to fit in”	2/4 groups expressed dissatisfaction with amount of content:<br>e.g. “There was a lot of course content to deliver in one session… babies needs are priority to mother and too much information to take in.
PN3	4	3.50 (0.58)	4.25 (0.96)	2/4 groups reported minor changes to content:<br>Groups A and D/E omitted ages and stages quiz, and feelings	2/4 Groups reported problems with timings due to late arrivals or entertaining babies<br>e.g. “The timings were not good, trying to get all the information to parents around feeding baby, nappy changing, sickness was distracting mothers”	2/4 Groups reported problems with materials<br>e.g. “too many hand-outs to look through”<br><br>“Pedometer challenge was confusing. No answer for what prize will be, how steps are converted to miles.”<br><br>“I have some confusion about serving sizes - 'what is a

Session	Number of logs received	Ease of delivery: Mean (SD)[a]	Participants engaged? Mean (SD)[b]	Any change to content?	Comments on timings	Notes about materials
						portion', 'when they are full'
PN4	2*	5.00<br>SD (0)	5.00 (SD 0)	1/2 groups reported minor changes to content<br>Group B: Changed the way delivered family food section - didn't have flipcharts on wall, but just had discussion	No problems with timings were reported	No problems with materials were reported
PN5	2*	4.00 (SD 0)	5.00 (SD 0)	1 group reported minor change in content<br>Group B: changed order of activities to suit needs of group	1 group reported being rushed due to late arriving families	One group reported that 'activities section' needs to be clearer

[a] scored on a 1-5 scale where 1 – complicated / challenging to 5 – straight forward / easy

[b] scored on a 1-5 scale where 1 – not at all to 5 – very receptive

NB: AN: Antenatal; PN: Postnatal. Only one log for postnatal session 6 was completed. This is not displayed.
