# Supplementary material for: The HAPPY (Healthy and Active Parenting Programmme for early Years) feasibility randomised control trial: acceptability and feasibility of an intervention to reduce infant obesity
Source: BMC Public Health. 2016 Mar 1;16:211. doi: 10.1186/s12889-016-2861-z (PMC4774160; doi:10.1186/s12889-016-2861-z)
Supplement: Additional file 6: — Summary of resources required to deliver the intervention – Table of intervention costs. (PDF 272 kb) [file 12889_2016_2861_MOESM6_ESM.pdf]

Additional file 6: Summary of resources required to deliver the intervention for antenatal and postnatal sessions

	Per participant		Per group	
Antenatal	Plastic folder	£2.00	2 manuals with hand-outs	£10.00
	Memory book (blank notebook)	£1.00	Ring-binder for manual	£2.00
	Pen	£0.20	3 A0 size (large poster size) display boards for each group	£69.00
	The Parenting Puzzle book	£12.00	4 constructs board	£23.00
	Tommy's leaflet: Managing your weight in pregnancy	£0.00	The Nurturing Game	£23.00
	Start4Life Off to the Best Start booklet	£0.00	Why Love Matters – Sue Gerhard	£11.69
	Session 2: 'What we pay attention to' magnet	£2.80	Baby naming dictionary	£7.00
	Session 3: 'Praise is magic' magnet	£2.80	The Social Baby-Lynne Murray	£9.65
	Session 4: Candle & candle holder	£2.00	The Social Baby DVD - Lynne Murray	£19.00
	Session 5: 'Have I nurtured myself?' magnet	£2.80	Table cloth	£1.25
	Session 6: Gift cards (5 per participant + spares)	£0.03	Flowers for each session (optional)	£17.94
	Session 6: Gift boxes (1 per participant)	£0.40	Name label badges	£2.50
	Session 6: attendance certificate printed on card	£0.03	Set of felt tips	£1.50
			Flip chart easel	£33.67
			Flip chart paper	£6.46
			Flip chart pens	£1.74
			Set of post-it notes	£2.85
			Ball or bean bag	£1.50
			Mints for participants for each session	£6.00
			Dice	£1.80
			Water jug and glasses	£18.00
			Red, green, orange slips of paper	£2.80
			Ball of string	£2.99
			Set of ping pong balls and marbles of different sizes	£4.65
	Subtotal Antenatal	***£26.06***		***£279.99***

	Per participant		Per group	
Postnatal	Plastic folder	£2.00	2 manuals with hand-outs	£10.00
	Now I can crawl booklet	£1.12	Ring-binder for manual	£2.00
	Pen	£0.20	Photography	£50.00
	Session 3: pedometer	£1.00	Re-use group resources from antenatal classes except for mints, flowers, flip chart paper	£30.00
	Session 3: option to give small gift to participants	£2.00	Tape	£1.50
	Session 4: gift for baby such as plastic feeding bowl	£1.00	Popcorn kernels	£1.00
	Session 5: option to give small gift to participants	£2.00	Baby Sam toy	£10.00
			Baby mat or towel	£5.00
			Card for lollipop shapes	£2.60
			Types of boundaries board	£23.00
			Baby toys and balls	£10.00
	Subtotal Postnatal	***£9.32***		***£145.10***
	**TOTAL PER PARTICIPANT**	**£35.38**	**TOTAL PER GROUP**	**£425.09**
